# Soil Bacterial Community May Offer Solutions for Ginger Cultivation

**DOI:** 10.1128/spectrum.01803-22

**Published:** 2022-09-13

**Authors:** Chih-Wei Wang, Jing-Wen Michelle Wong, Shu-Shuo Yeh, Yunli Eric Hsieh, Ching-Hung Tseng, Shan-Hua Yang, Sen-Lin Tang

**Affiliations:** a Taitung District Agricultural Research and Extension Station, Council of Agriculture, Executive Yuan, Taitung, Taiwan; b Biodiversity Research Center, Academia Sinica, Taipei, Taiwan; c Molecular and Biological Agricultural Sciences, Taiwan International Graduate Program, Academia Sinica, Taipei, Taiwan; d Graduate Institute of Biotechnology, National Chung Hsing University, Taichung, Taiwan; e Institute of Fisheries Science, National Taiwan University, Taipei, Taiwan; f Bioinformatics, Institute of Biochemistry and Biology, University of Potsdam, Potsdam, Germany; g Systems Biology and Mathematical Modelling, Max Planck Institute of Molecular Plant Physiology, Potsdam, Germany; h Germark Biotechnology Co., Ltd., Taichung, Taiwan; i Biotechnology Center, National Chung Hsing University, Taichung, Taiwan; USDA – San Joaquin Valley Agricultural Sciences Center

**Keywords:** *Bacillus* *velezensis*, Etridiazole, ginger, soil microbiome

## Abstract

The Taitung region is one of Taiwan’s main sites for ginger agriculture. Due to issues with disease and nutrients, farmers cannot use continuous cropping techniques on ginger, meaning that the ginger industry is constantly searching for new land. Continuous cropping increases the risk of infection by Pythium myriotylum and Ralstonia solanacearum, which cause soft rot disease and bacterial wilt, respectively. In addition, fertilizer additives, which are commonly used to increase trace elements in the soil, cannot restore the soil when it is undergoing continuous cropping on ginger, even when there has been no observable decrease in trace elements in the soil. Recent studies about soil microbiome manipulation and the application of microorganisms have shown that plant-associated microbes have the ability to improve plant growth and facilitate sustainable agriculture, but studies of this kind still need to be carried out on ginger cultivation. Therefore, in this study, we used the bacterial 16S V3–V4 hypervariable region of the 16S rRNA region to investigate microbe compositions in ginger soil to identify the difference between ginger soil with and without disease. Later, to investigate the influence of the well-known biocontrol agent *B. velezensis* and the fungicide Etridiazole on soil microbes and ginger productivity, we designed an experiment that collected the soil samples according to the different periods of ginger cultivation to examine the microbial community dynamics in the rhizome and bulk soil. We demonstrated that *B*. *velezensis* is beneficial to ginger reproduction. In accordance with our results, we suggest that *B. velezensis* may influence the plant’s growth by adjusting its soil microbial composition. Etridiazole, on the other hand, may have some side effects on the ginger or beneficial bacteria in the soils that inhibit ginger reproduction.

**IMPORTANCE** *Pythium myriotylum* and Ralstonia solanacearum cause soft rot disease and bacterial wilt, respectively. In this study, we used the bacterial 16S V3–V4 hypervariable region of the 16S rRNA region to investigate microbe compositions in healthy and diseased ginger soil and find out the influence of the well-known biocontrol agent B. velezensis and the fungicide Etridiazole on soil microbes and ginger productivity. These results demonstrated that *B*. *velezensis* benefits ginger reproduction and may influence the soil bacterial composition, while Etridiazole may have some side effects on the ginger or beneficial bacteria in the soils. The interactions among ginger, biocontrol agents, and fungicides need to be further investigated.

## INTRODUCTION

Ginger (Zingiber officinale) is an herbaceous perennial with underground rhizomes that is widely used as a fresh vegetable, spice, and herbal medicine. Its benefits include stimulating appetite, improving gastrointestinal motility, encouraging sweating, promoting a healthy stomach, reducing cold symptoms, refreshing the mind, and reducing unpleasant seafood odors to enhance the flavor and aroma of seafood cuisine. According to statistics from the Taiwan Agriculture and Food Agency, Council of Agriculture, Executive Yuan (AFA) (https://data.gov.tw/license), Taitung County has more than 200 ha of land used for ginger farming, making it the second-largest ginger-producing area in Taiwan.

The major diseases that threaten ginger production include soft rot disease caused by *Pythium myriotylum* and ginger bacterial wilt caused by the bacterial pathogen Ralstonia solanacearum. Soft rot disease tends to be prevalent during the summer months, as *P. myriotylum* prefers warmer weather and wet soil conditions (1). *P. myriotylum* produces numerous mobile spores, moves through flowing water from rainfall or irrigation, and spreads rapidly from infected regions to the entire ginger plant. According to a report by Stirling et al. (2), it can take as little as 2 months for soft rot disease to infect the entire plant and completely destroy a harvest. The transmission of bacterial wilt is similar to soft rot disease, but takes longer to progress and may occur at any time during the growth period, causing huge losses in yield.

The first step in preventing these diseases is to get healthy, pathogen-free seeds. Ginger reproduces asexually, using the rhizomes harvested from the previous farming period as the mother rhizomes. These mother rhizomes are then cut into pieces to make seed rhizomes. Experienced farmers will choose the mother rhizomes collected in the previous year from disease-free land. In addition, most farmers need to find new land that has never had ginger planted in it to reduce the risk of disease. However, land that is suitable for ginger is becoming more and more difficult to find, and farmers will sometimes farm illegally in forests or developed land, affecting soil and water conservation and homeland security. Moreover, once disease becomes widespread, the consequent reduction in ginger yield can cause serious economic losses. For example, *Pythium myriotylum* caused a 20% decrease in the annual yield of ginger in Taiwan and destroyed the infected yield within a week (1). This is a major challenge in ginger agriculture that must be overcome.

According to personal communications with ginger farmers, this problem of ginger crop reduction cannot be improved with fertilizer. Soil analysis showed no significant changes in the concentration of microelements (C. W. Wang, unpublished data), meaning that there are other factors that lead to reductions in yield. Researchers speculate that long-term applications of chemical pesticides and fertilizers change the soil microbiome, prevent the continuous cropping of ginger, and promote disease occurrence, but the exact cause of the ginger cultivation issue is still unknown. Previous studies on ginger explored ways to promote ginger growth by investigating the plant’s physiological properties and revealing the biosynthetic pathway of bioactive compounds and their benefits to human health. However, little is understood about the obstacles to ginger cropping or the plant’s soil microbiome.

Recently, fast, cost-efficient, and convenient next-generation sequencing (NGS) technology has made it easier to explore genome sequences and reveal gene expressions responsible for the biosynthesis of bioactive compounds in ginger. Studies on the ginger rhizome based on *de novo* transcriptome analysis, genome sequencing, and metabolomic analysis have provided molecular information on bioactive compounds stored in ginger rhizomes such as gingerol, volatile oil, and diarylheptanoids (3), and have identified the 12 enzymatic gene families that are involved in the biosynthesis of gingerol (4). Furthermore, according to studies from India, comparative transcriptome analyses of Zingiber officinale Rosc. and Curcuma amada Roxb. have yielded information about genes related to the resistance mechanism against bacterial wilt infection (5) as well as the effect of different agro-climatic conditions on ginger’s secondary metabolism expression (6).

In addition, plant growth-promoting rhizobacteria (PGPR), such as the Gram-positive *Bacillus* species, are widely used in agriculture because they are associated with many crops and form an endospore during hot and dry conditions (7). *B. velezensis* is a member of the genus *Bacillus* and acts as a powerful biocontrol agent in agriculture. It has been used as a biocontrol agent against Ralstonia solanacearum, which causes tomato and banana wilt disease (8). Although *B. velezensis* has been widely used to promote plant growth, whether it can improve ginger growth or change the root-associated microbiome is still unknown.

Although there have been many studies on the microbial compositions of crops, the difference between a healthy and diseased ginger soil microbiome remains unclear. How chemicals and biocontrol agents affect the dynamics in the soil microbiome of ginger is also unknown. Therefore, there are two parts to this study. In the first part, we compared the microbial composition in soils of healthy ginger and diseased ginger to understand the microbial community dynamics in the soil close to the ginger roots (rhizosphere-detritusphere habitats) and the bulk soil. In the second part, to investigate the influence of PGPR and fungicide on the soil microbes and productivity of ginger, we designed an experiment that collected soil samples according to the different ginger cultivation periods to examine the microbial community dynamics in the soil close to the ginger rhizomes and the nearby soil after adding *B. velezensis* or the fungicide Etridiazole.

## RESULTS

### Comparison of diseased and healthy soils.

**FIG 1:**
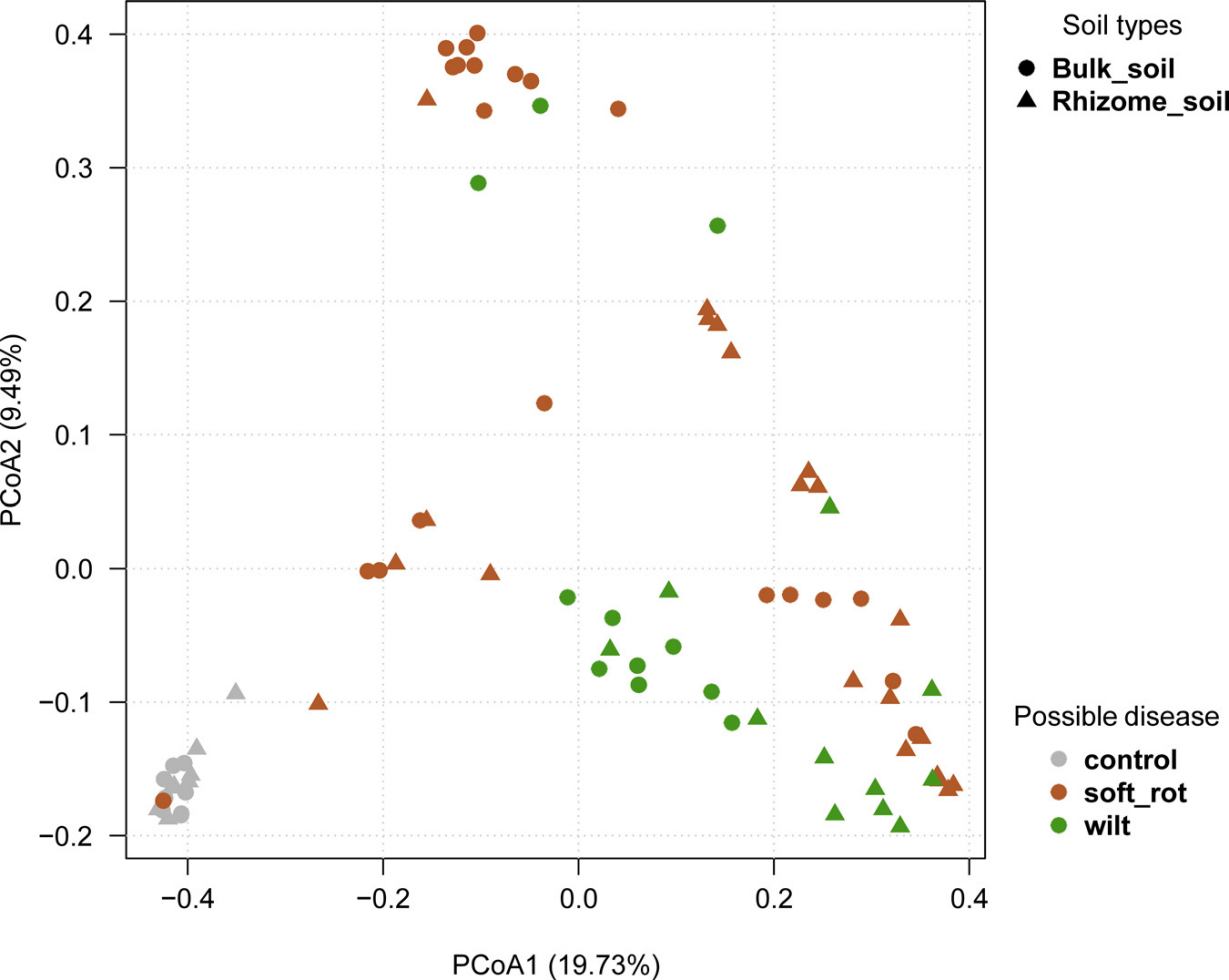
Principal coordinates analysis (PCoA) of bacterial communities from the bulk and rhizome parts of healthy soil, soft rot disease soil, and ginger bacterial wilt soil. The shapes indicate bulk and rhizome soils, and the colors indicate healthy (control) and diseased (soft rot disease soil and bacterial wilt soil) soils in the rhizome soil, and the bacterial compositions of three different soil samples are significantly different (ANOSIM *R* = 0.357, *P* = 0.001), while the bacterial composition of the bulk soil has only minor differences (ANOSIM *R* = 0.261, *P* = 0.001).

We observed that the bacterial composition was distinct among healthy soil, soft rot disease soil, and ginger bacterial wilt soil samples (Fig. 1; analysis of similarity [ANOSIM] *R* = 0.306, *P* = 0.001). When focusing on the rhizome soil, the bacterial compositions of three different soil samples were significantly different (ANOSIM *R* = 0.357, *P* = 0.001), while the bacterial composition of the bulk soil had only minor differences (ANOSIM *R* = 0.261, *P* = 0.001) compared to that of the rhizome soil. In healthy soil, remarkable similarities were found between the bacterial compositions of the bulk and rhizome soils. In diseased soil, however, the bacterial compositions of the bulk and rhizome soils were different (Fig. 1), showing that the microbial composition of bulk and rhizome soils of ginger changed after being infected.

**FIG 2:**
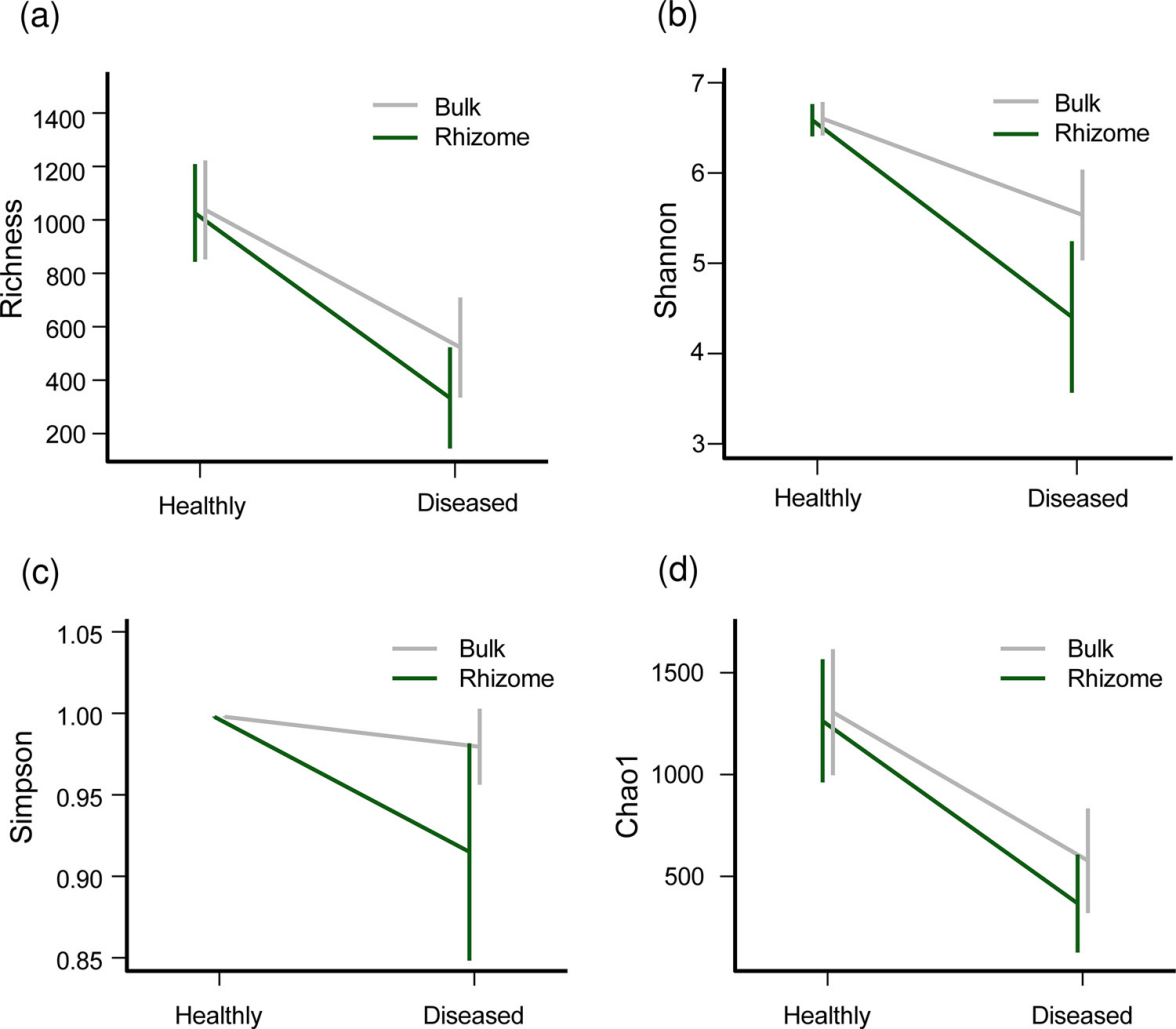
The difference in bacterial composition in both the bulk and rhizome parts of healthy and diseased soils, according to the alpha diversity indices. (a) Richness index; (b) Shannon index; (c) Simpson index; and (d) Chao1 index. The gray color indicates the samples of bulk soil, and the green color indicates the samples of rhizome soil. In the diseased soil, the Shannon and Simpson index values of the rhizome soil drop significantly more than in the bulk soil (two-way ANOVA of Shannon index, F = 10.01, *P* = 0.002; two-way ANOVA of Simpson index, F = 6,548, *P* = 0.012).

The rhizome soil had lower bacterial diversity than the bulk part in the healthy soil, but the difference was not significant (*t* test in Simpson, *P* = 0.6809; in Shannon, *P* = 0.864; in Richness, *P* = 0.8982; in Chao1, *P* = 0.7853; Fig. S1 in the supplemental material). However, in the diseased soil, the Shannon and Simpson index values of the rhizome soil dropped significantly more than the bulk soil (two-way ANOVA of Shannon index, F = 10.01, *P* = 0.002; two-way ANOVA of Simpson index, F = 6,548, *P* = 0.012) (Fig. 2); the mean Shannon index in healthy rhizome was 6.587, but in soft rot disease rhizome and wilt rhizome was 4.531 and 4.172, respectively; and the mean Simpson index in healthy rhizome was 0.998, but in soft rot disease rhizome and wilt rhizome were 0.924 and 0.898, respectively. The results suggest that bacterial diversity decreased after the ginger was infected and that the infected area was mainly limited to the soil that the ginger root touched. Fig. 1 and Fig. S1 show that the bacterial composition of the ginger rhizome part changed, and that the rhizome part’s bacterial diversity was lower than the bulk part’s.

**FIG 3:**
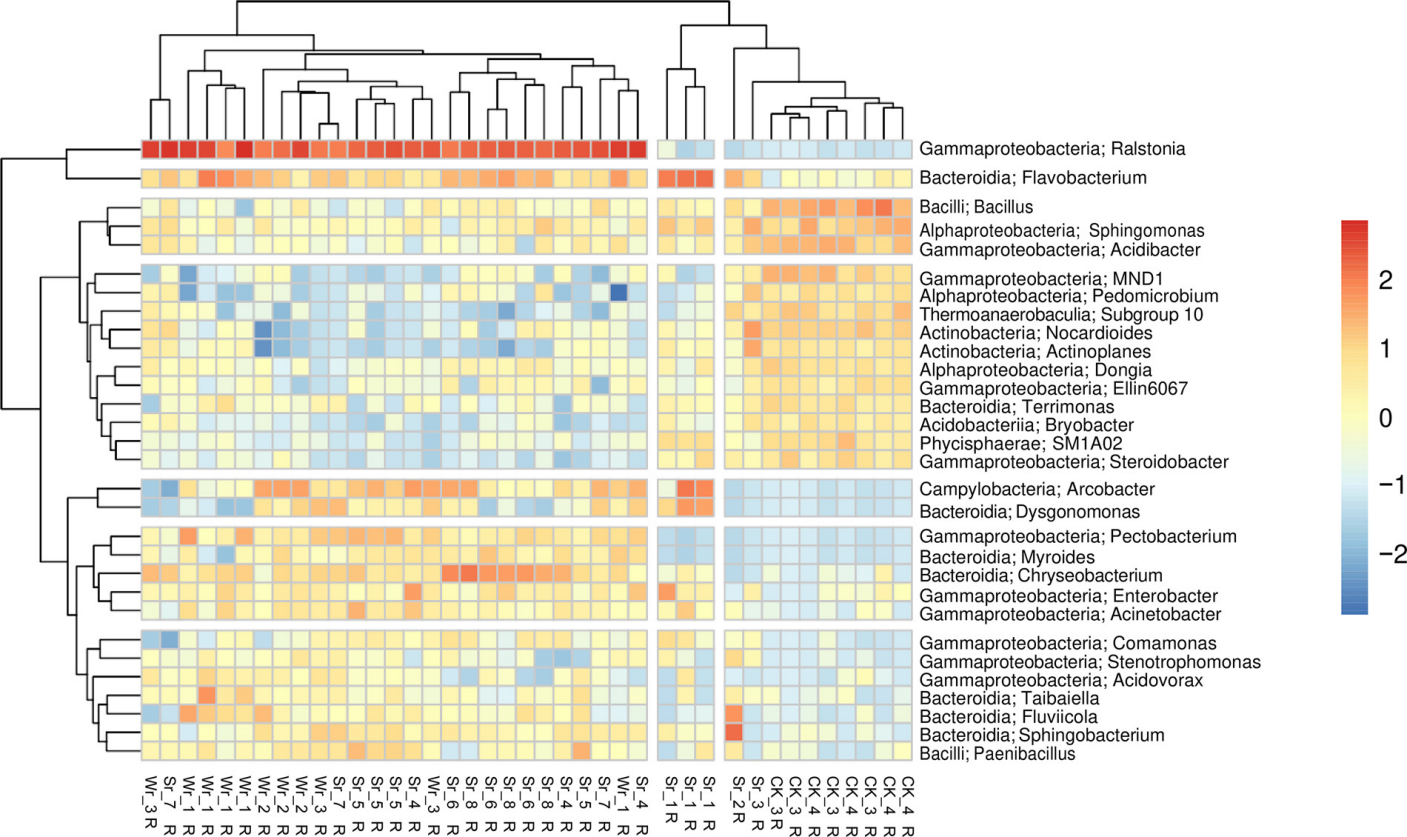
Heatmap of two-way clustering of the top 30 genera in the rhizome part of healthy and diseased soils. The colors indicate relative abundance: warmer colors (yellow to red) indicate higher abundances and cooler colors (blues) indicate lower abundances. CK indicates control soil samples, Sr is soil with soft rot disease, and Wr is soil with wilt disease. Both ginger bacterial wilt soil and soft rot disease soil have remarkably different bacterial communities compared to the control soil.

The 30 most abundant bacterial genera (the top 30 genera) in the rhizome parts of the healthy and diseased soils showed that both ginger bacterial wilt soil and soft rot disease soil had remarkably different bacterial compositions compared to the control soil (Fig. 3). In the diseased soil samples, all the ginger bacterial wilt soils and most of the soft rot disease soils were dominated by the genus Ralstonia. The relative abundance of *Ralstonia* was low in healthy soil. Arcobacter, Dysgonomonas, Pectobacterium, and Myroides were only found in diseased soil. Some bacteria were present in both the healthy soil and the diseased soil, but had a higher relative abundance in diseased soil, such as *Flavobacterium*, *Chryseobacterium*, Enterobacter, Acinetobacter, Comamonas, Stenotrophomonas, Acidovorax, Taibaiella, Fluviicola, Sphingobacterium, and Paenibacillus. Additionally, relative abundances of *Bacillus*, Sphingomonas, Acidibacter, Nitrosomonadaceae, Pedomicrobium, Thermoanaerobaculaceae, Nocardioides, Actinoplanes, Dongia, Terrimonas, Bryobacter, Phycisphaeraceae, and Steroidobacter were greater in healthy soil than in diseased soil (Fig. 3).

### Treatment experiment using *B. velezensis* or fungicide Etridiazole.

**FIG 4:**
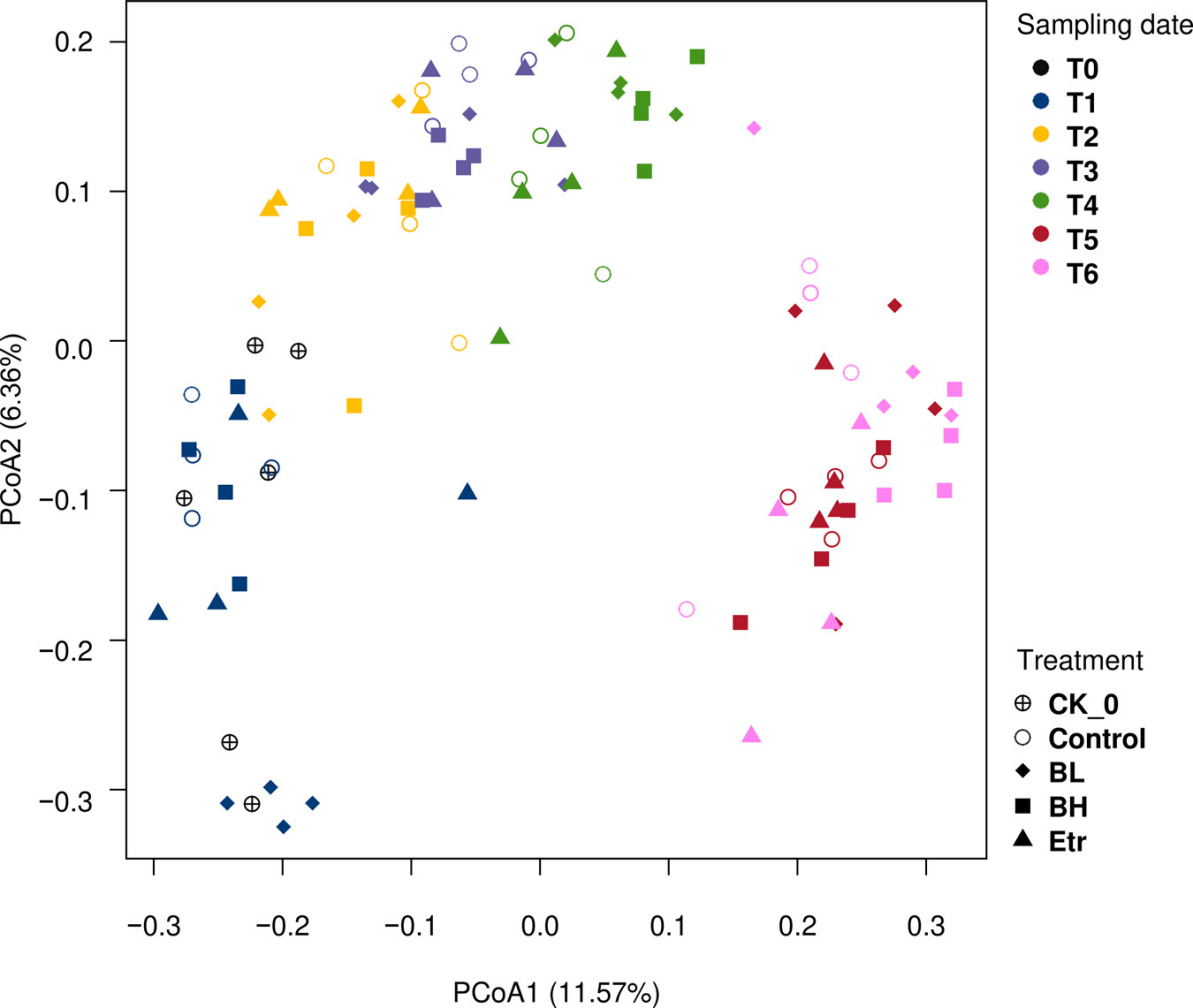
PCoA of bacterial communities from rhizome soils with different treatments along sampling times. The shapes and colors indicate different treatments and sampling times, respectively. CK_0 indicates the soil before the experiment began. Control indicates ginger soil without any treatment, BL is low amounts of *B. velezensis*, BH is high amounts of *B. velezensis*, and Etr is the Etridiazole treatment. Based on the results, time is the major factor influencing the bacterial composition, but the effects are unequal in different treatments.

**FIG 5:**
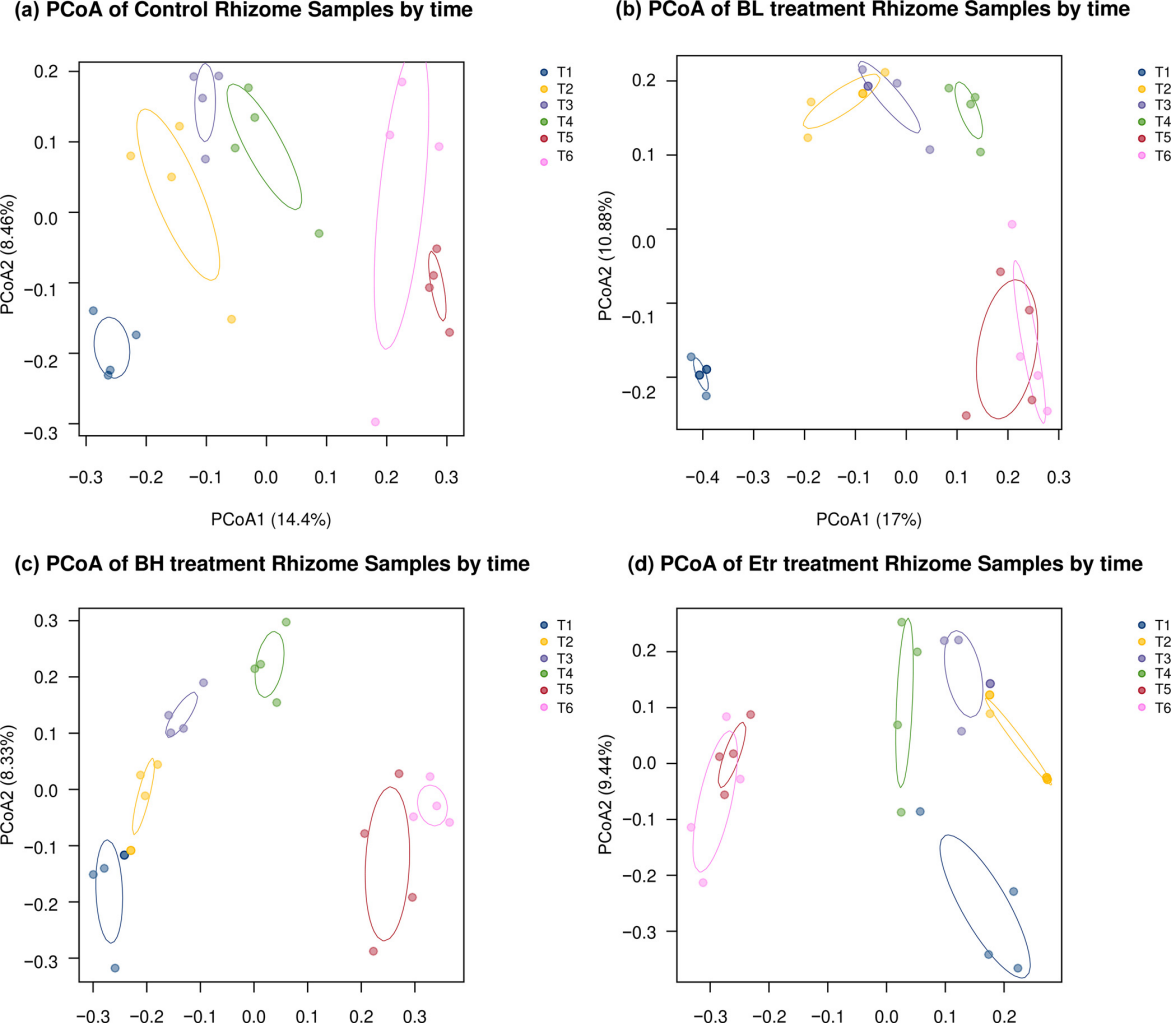
PCoA of bacterial communities from rhizomes in different treatments. (a) The control rhizome sample; (b) the BL treatment rhizome sample; (c) the BH treatment rhizome sample; and (d) the Etr treatment rhizome sample. Colors indicate the different time points. Bacterial composition of the rhizome part of each treatment changes over time (ANOSIM: in control, *R* = 0.637, *P* = 0.001; in BL, *R* = 0.795, *P* = 0.001; in BH, *R* = 0.728, *P* = 0.001; in Etr, *R* = 0.687, *P* = 0.001).

Comparing all the results of each treatment in the dosing test revealed that the bacterial composition changed over time (Fig. 4). Each group. including the control group, changed over time. At the final time point, the Etridiazole (Etr) group was distinct from the other treatment groups (Fig. 4). Based on the results, time was the major factor influencing the bacterial composition, but the effects varied in different treatments. Since the diversity index results showed that the bacterial composition of the rhizome part changed greatly, we analyzed the change in the bacterial composition of the rhizome part in each treatment group. Our analysis revealed that the bacterial composition of the rhizome part of each treatment group changed over time (Fig. 5; ANOSIM: in control, *R* = 0.637, *P* = 0.001; in the low-concentration *B. velezensis* treatment group (BL), *R* = 0.795, *P* = 0.001; in the high-concentration *B. velezensis* treatment group (BH), *R* = 0.728, *P* = 0.001; in Etr, *R* = 0.687, *P* = 0.001).

**FIG 6:**
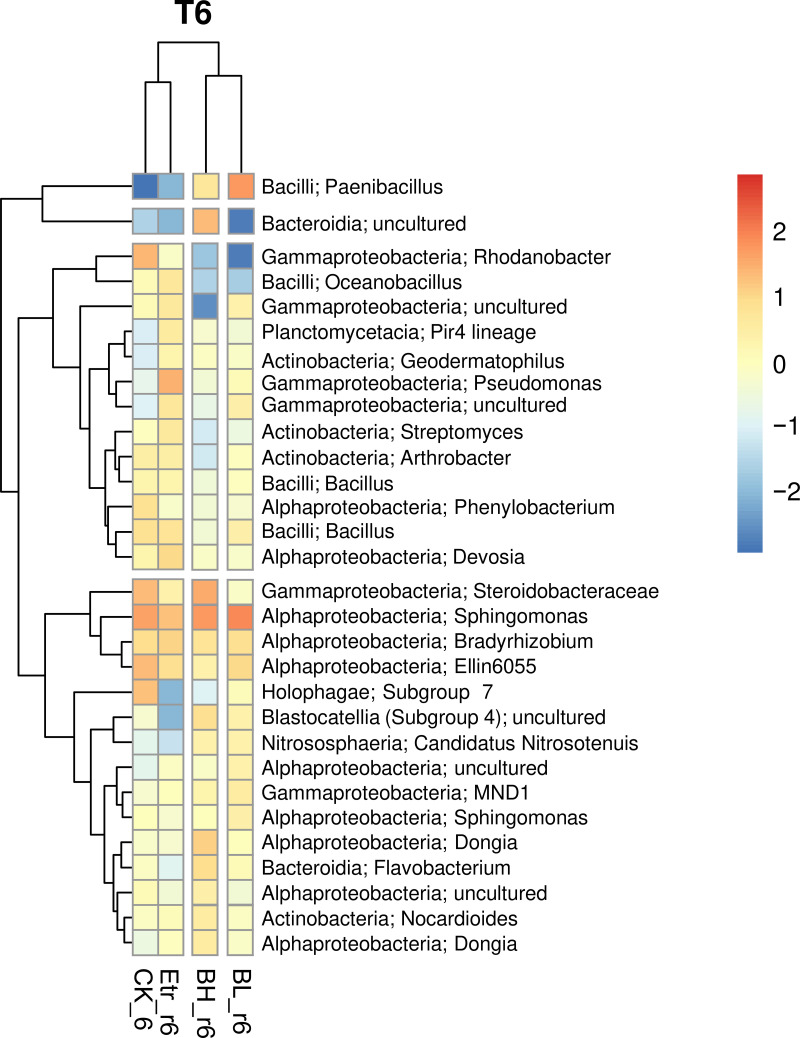
The relative abundance of the top 30 ASVs in the rhizome parts for four treatments in the last sampling time. CK indicates ginger soil without any treatment, BL is low amounts of *B. velezensis*, BH is high amounts of *B. velezensis*, and Etr is the Etridiazole treatment. BH and BL groups are clustered, and the Etr group are clustered with the control group.

Based on the 30 most abundant amplicon sequence variants (ASVs) in the rhizome part of each treatment group at every time point, we observed that the dominant bacterial composition changed continually (Fig. S2). At different time points, we observed a remarkable similarity between the BH and BL bacterial compositions, and a similarity between the Etr group and control group (CK) bacterial compositions. This situation continued until the final time point (Fig. 6), when the BH and BL groups were clustered and the Etr group was clustered with the control group.

At the first time point, *Bacillus* was found in four groups and was solely dominant in nonbacterial treatment groups; at the second time point, the relative abundance of Pseudomonas had increased to become the dominant genus with *Bacillus*; at the third time point, *Bacillus* remained a higher relative abundance genus; and at the fourth time point, the relative abundances of Sphingomonas and *Paenibacillus* increased beyond that of *Bacillus*. *Sphingomonas* was found in all four groups while, *Paenibacillus* was only observed in the bacterial treatment group. At the fifth time point, the bacterial treatment groups had greater relative abundances of *Bacillus* and *Sphingomonas* than did the control and Etr groups; at the final time point, the bacterial treatment groups were dominated by *Bacillus* and *Sphingomonas*. In addition, Pseudomonas was solely dominant in the Etr group at every time point.

**FIG 7:**
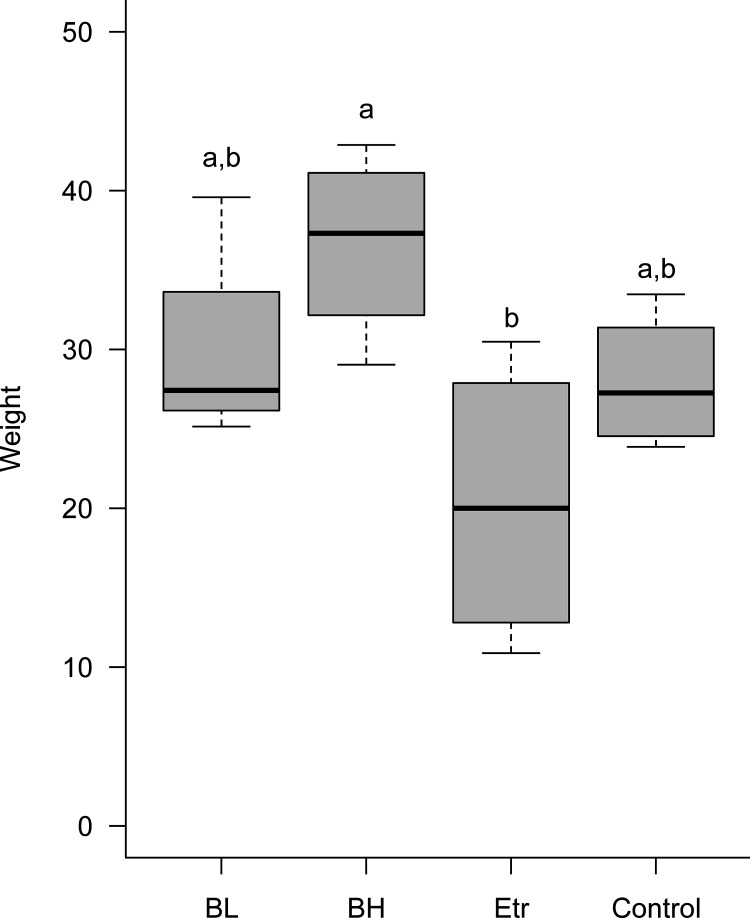
Production of ginger after different treatments. Control indicates ginger soil without any treatment, BL is low amounts of *B. velezensis*, BH is high amounts of *B. velezensis*, and Etr is the Etridiazole treatment. Ginger production is highest in the high-concentration BV treatment group (BH) and lowest in the Etr group. A Kruskal-Wallis test and Dunn’s *post hoc* test are used for all statistical analyses of group comparisons with a significance level of α = 0.05.

Ginger production was highest in the high-concentration *B. velezensis* (BV) treatment group (BH) and lowest in the Etr group (Fig. 7, Table S1). Although neither of the groups were significantly different from the control, they were significantly different to one another.

To determine the relative abundance of *B. velezensis* (BV138) in the bacterial composition after adding it to the soil, we further compared all the sequences of *Bacillus* contained in the soil samples. The database contains 69 ASVs that were *Bacillus*, and only three that were identified as *B. velezensis* (similarity of about 99%). These *B. velezensis* sequences were found at every time point among the BH samples, and its average relative abundance was 0.24%. In the BL group, *B. velezensis* was mainly found from the third to the sixth time point of partial samples, and its average relative abundance was merely 0.03%. There were no *B. velezensis* sequences in the control or Etr group. The results indicate that *B. velezensis* (BV138) relative abundances in soil samples were positively correlated with its added amount, which is dose dependent.

## DISCUSSION

Ginger is an important crop, but little research has been done on it compared to other agricultural plants. In the present study, we investigated the bacterial compositions in the rhizome and bulk regions of both healthy and diseased soils that previously grew ginger. We found that bacterial diversity was lower in diseased soil than in healthy soil, a phenomenon that was magnified in the rhizome region. Generally, the soil microbial community is influenced by plant growth because of the chemicals released by plant roots (9). Therefore, microbial diversity decreased in the rhizome, but microbial biomass increased in bulk soils (9). A review from Liu et al. (10) showed that plants can search for microbial assistance to resist pathogens, and thus, the microbial community dynamics may be caused by the diseased plant.

In our study, based on the 30 most abundant ASVs, bacterial composition in healthy and diseased rhizomes was significantly different. In addition to the pathogen R. solanacearum, the rhizome of the diseased soils showed an increase in the relative abundance of some bacteria. Members of Flavobacterium and Chitinophagaceae had higher relative abundances in diseased soil than healthy soil. Members of *Flavobacterium* are usually found in the rhizosphere with high abundance and have been thought to play a role in protecting plants from disease (11). A recent report indicated that *Chitinophaga* and *Flavobacterium* in endophytic bacterial communities may have the ability to suppress pathogens from soil (11). For example, sugar beet roots can attract *Chitinophaga* and *Flavobacterium* into the endosphere to suppress the fungal pathogen R. solanacearum (12). Although our study did not investigate endophytic bacteria, the relative abundance of *Flavobacterium* did increase with that of *Ralstonia*, indicating that *Flavobacterium* may suppress Ralstonia in the root area of ginger. In addition, some species in Stenotrophomonas and Sphingobacterium were found to play a role in inhibiting the growth and virulence of plant pathogens and have the ability to rescue plants from stresses (13, 14). Both genera had higher relative abundances in the diseased soil than healthy soil in the present study. However, it is not clear if their abundance increased because of the plant host becoming infected.

*Bacillus*, *Sphingomonas*, and *Acidibacter* were constantly present in both healthy and diseased soil but had higher relative abundance in the healthy soil. Although it is unclear why these bacterial genera were present in both healthy and diseased soil, previous studies have shown that *Bacillus*, *Sphingomonas*, and *Acidibacter* are beneficial bacterial groups that promote plant growth (15 to 17).

Bacterial strains have been used as biofertilizers to ameliorate plant production, and chemicals have been used as pesticides and fungicides to maintain plant health. In this study, we found that applying the bacterial strain *B. velezensis* and the fungicide Etridiazole can decrease bacterial compositions in soil, especially in the rhizome part. This phenomenon is mainly constrained to the rhizome, which comes in direct contact with the ginger root, indicating that ginger may influence the bacterial composition during the treatments. As we discussed previously, plant roots may release some chemicals to adjust the soil microbial community (9).

However, in this study, we found that using Etridiazole resulted in the largest change in bacterial composition. Etridiazole (5-ethoxy-3(trichloromethyl)-1,2,4-thiadiazole) causes the hydrolysis of cell membrane phospholipids into free fatty acids and lysophosphatides, leading to the lysis of membranes in fungi. Therefore, it has been used as a fungicide. In addition to damaging fungi, Etridiazole has side effects on other soil microorganisms because it reduces the nitrification rate of ammonium-oxidizing bacteria in soil, which may change the soil microbial community and influence the soil structure and function (18, 19).

In this study, Pseudomonas and *Bacillus* had higher relative abundances in the treatment with Etridiazole. According to Shen et al. (2019), some rice endophytes, such as B. aryabhattai and P. granadensis, can tolerate two or more fungicides, including Etridiazole. In addition, they found that some strains may fix nitrogen, solubilize phosphorus, and produce indole acetic acid (IAA), which may promote plant growth and is believed to be a biofertilizer for rice (20). Here, we suggest that Pseudomonas and *Bacillus* show a similar tolerance to Etridiazole when it is used on ginger. Hence, although we did not treat the ginger with Etridiazole and *B*. *velezensis* together, based on the dominant bacterial genera in the soil with Etridiazole, we suggest that Pseudomonas and *Bacillus* could be bacterial biofertilizers for ginger when used with the fungicide Etridiazole.

*Bacillus* species are PGPR that can survive even when their endospores are converted into a dry powder to preserve them for a long time. The application of spore-forming *Bacillus* spp. does not have a lasting effect on the composition of the rhizosphere bacterial community (21). In this study, *B*. *velezensis* changed the bacterial compositions in soil, but *B*. *velezensis* also increased the production of ginger in a dose-dependent manner.

The microbial defense mechanisms of *B. velezensis* have been studied in other plants. For example, *B. velezensis* FZB42 produces bioactive molecules that are active against microorganisms (22), including surfactin, iturin, and fengycin—all of which are antifungal, lipopeptide compounds (23). Moreover, the antibacterial compounds difficidin and bacilysin are also produced by *B. velezensis* and are responsible for antagonistic activity against Xanthomonas oryzae pv. *oryzae* and *X. oryzae* pv. *oryzicola*, which cause rice diseases such as bacterial blight and bacterial leaf streak (24). *B. velezensis* also synthesizes plantazolicin, which kills parasitic nematodes (25). Furthermore, it was found that the biofilm formed by *B. velezensis* in plant rhizospheres can promote plant growth and secrete antimicrobial compounds to resist the invasion of infectious microbes (26).

Here, although the mechanisms are not yet clear, we found that the relative abundances of *B*. *velezensis* in samples were not high. We suggest that *B*. *velezensis* does not improve the ginger production by itself directly, but instead may influence ginger production indirectly by adjusting the bacterial community gradually. Bacteria belonging to the genus Paenibacillus have been isolated from diverse environments, especially from soils. Many of them ameliorate crop growth via biological nitrogen fixation, phosphate solubilization, production of phytohormone indole-3-acetic acid (IAA), and the emission of siderophores to increase iron acquisition (27). For example, Paenibacillus jamilae HS-26, which synthesizes hydrolytic enzymes and releases extracellular antifungal metabolites and volatile organic compounds—primarily, N, N-diethyl-1, 4-phenylenediamine—has highly antagonistic activity against several soilborne pathogens (28). Some *Paenibacillus* species, such as P. macerans, are used in commercial biofertilizers, but their performance may be limited by soil pH, salinity, moisture content, and temperature (29).

In this study, the relative abundance of *Paenibacillus* and *Bacillus* increased in the treatments with *B*. *velezensis*. Some *Bacillus* and *Paenibacillus* can elicit induced systemic resistance (ISR), similar to members of Pseudomonas, stimulating plant defense mechanisms against pathogens (30). Therefore, *Bacillus*, *Paenibacillus*, and Pseudomonas may serve redundant functions in soil, which may explain why their relative abundances change so much.

In the soil microbiome, fungi are one of the important domains that influence plant and soil condition. Although we tried to investigate the fungal community through NGS methods, we encountered some limitations and thus did not find it necessary to provide the results here. For example, in the comparison of diseased and healthy soils, we found that the fungal compositions of three different soils were significantly different, showing a similar pattern to the bacterial composition (data not shown). However, we found that our data of fungal composition were not complete because they lacked several groups of important plant pathogens, such as *Pythium*. The region we used for investigating fungal composition was the internal transcribed spacer (ITS) of the nuclear ribosomal RNA (rRNA), since most high-throughput sequencing (HTS)-based studies focus on either the ITS1 or ITS2 subregion (31). However, the ITS region provides insufficient resolution for species-level assignment (32), because its length is variable among different fungal genera and species (33, 34). In addition, lacking information of the whole ITS sequence of *Pythium* in the database makes it difficult to identify the taxa for this study. Hence, we did not find *Pythium* in our result. Furthermore, previous studies addressing fungi usually used the small subunit (SSU) (18S) and large subunit (LSU) (28S) nuclear rRNA genes as marker genes. However, for ascomycetes and basidiomycetes, these markers are often only informative on taxonomic levels above species or genera because there may be no or too little variation in SSU and LSU sequences to detect difference among species (31). Also, some studies selected the D1/D2 region of LSU to identify fungal taxa by 454 pyrosequencing or sanger sequencing, but this region is too long (>500 bases) for an HTS-based method, such as the Illumina sequencing used in this study. Therefore, we suggest that the third-generation techniques using Pacific Biosciences (PacBio) and Oxford Nanopore platforms can be used for soil fungal dynamics in the future.

Although we did not study the function of *B. velezensis* in ginger disease, we demonstrated that *B*. *velezensis* benefits ginger reproduction. We also suggest that *B. velezensis* may influence the plant by adjusting the soil bacterial composition. Regarding the function of Etridiazole in ginger reproduction, although treatment with Etridiazole did not significantly decrease the plant’s production, we suggest that Etridiazole may have some side effects on the ginger or beneficial bacteria in the soils that should be investigated further. Furthermore, whether using *B*. *velezensis* affects Etridiazole’s impact on reproduction is an interesting question that should be investigated. Our results provide clues for further investigations focusing on the interactions among the ginger, biocontrol agents, and fungicides.

## MATERIALS AND METHODS

### Collecting soil samples from diseased ginger.

The soils of 32 ginger plants showing yellowing and wilting symptoms were collected from 12 different ginger fields in Taitung County from 3 July to 21 October 2019. For each diseased ginger plant, the bulk soil and rhizome soil were collected. All of the soil samples were stored in a −80°C freezer until DNA extraction.

### Treatment experiment using *B. velezensis* and fungicide Etridiazole.

The experimental field (GPS: 22.940611˚N, 121.123861˚E) was located in Luye Township, Taitung County, Taiwan. Ginger plants were planted on 29 March 2019, using four treatments: BV138 200× dilution (BL group), BV138 25× dilution (BH group), Terrazole (containing 25% Etridiazole) 1,500× dilution (Etr group), and a control irrigated with water. Each treatment plot was 3 m long and 0.3 m wide, with two rows planted with 40 ginger seed rhizomes. Each treatment was conducted with four replicates and arranged by randomized complete block design (RCBD) (Fig. S3). The BV138 microbial reagent used in the experiment was isolated from the soil in Taitung and identified as Bacillus velezensis. The BV138 was manufactured by Yuan-Mei Biotech (Taichung, Taiwan) as powder containing 5 × 10^9^ CFU/g of *B. velezensis* cells. For each BV138 treatment, 12.5 L of a diluted reagent of *B. velezensis* cells was irrigated.

The reagents were added on 3 April, 2 May, 5 June, 18 June, 3 July, 18 July, 31 July, 16 August, 21 August, and 11 September 2019 (Fig. S4). When collecting soil samples, 40 ginger plants in each treatment were randomly selected, uprooted, and shaken vigorously to remove the soil attached to the rhizomes and roots. Soil was collected from the planting site down to 15 cm deep and labeled as bulk soil. The soil tightly attached to the rhizomes and roots was brushed off with a sterilized paintbrush and collected as the rhizome soil. All of the soil samples were stored in a −80°C freezer until DNA extraction. The soil samples were collected on 26 April, 3 June, 1 July, 26 August, and 13 November 2019, and 6 January 2020. Twelve soil samples collected from six randomly picked ginger rhizomes in the same field prior to irrigation on 3 April 2019, were defined as “Day 0.” The experimental field was managed regularly by a farmer with over 30 years of experience cultivating ginger. Herbicide was applied on 1 April 2019. Fungicide and pesticide were applied on 14 May, 30 June, 20 July, 23 August, 15 September, and 28 September 2019. Fertilizer was applied on 16 June, 26 July, 15 September, and 18 October 2019 (Fig. S4).

### DNA extraction, marker gene amplification, barcoding, and sequencing.

DNA extraction was performed using the DNeasy PowerSoil kit (Qiagen, MD, USA) according to the manufacturer’s protocol. For the bacterial composition survey, the V3–V4 hypervariable region of the 16S ribosomal RNA (rRNA) genes was amplified using PCR with the primers 341F (5′- CCTACGGGNGGCWGCAG-3′) and 805R (5′-GACTACHVGGGTATCTAATCC-3′). Subsequently, a DNA-tagging PCR (five cycles) was used to tag each of the PCR products (every six samples were tagged individually and mixed). The PCR products were run in 2% agarose gel (SeaKem LE Agarose, Lonza, ME, USA), purified with a MinElute Gel Extraction kit (Qiagen, Hilden, Germany), and quantified using a QuantiFluor dsDNA System (Promega Corporation, Madison, WI, USA) on a Qubit 2.0 Fluorometer (Invitrogen, Grand Island, NY). The paired-end library was constructed with a Celero DNA-Seq System (1-96) (Nugen, San Carlos, CA, USA); all procedures were in accordance with the manufacturers’ instructions. The library concentration and quality were assessed on a Bioanalyzer 2100 (Agilent Technologies, Santa Clara, CA, USA) using a DNA 1000 lab chip (Agilent Technologies). 16S amplicon libraries were sequenced 2 × 301 + 16 bp (dual index) using a Miseq reagent kit v3 (600 cycles) on an Illumina MiSeq system. PCR, barcoding, and sequencing experiments were performed by Tri-I Biotech (New Taipei City, Taiwan). All of the bacterial community sequences were deposited into GenBank (SRA accession: PRJNA826673).

### Bioinformatic analyses and statistics.

The 16S rRNA gene amplicon sequences were processed using the Quantitative Insights Into Microbial Ecology 2 (QIIME 2) pipeline (version 2019.10) (35). The raw reads were flipped into the same orientation by Cutadapt (version 1.15) (36), truncated at 235 bp at both ends, and denoised using the DADA2 plugin of QIIME2 (37). The amplicon sequence variants (ASVs) were obtained via the denoising process with quality filtering and chimera removal. ASV taxonomy was assigned using the classifier-consensus-vsearch plugin (38) against SILVA NR132 99% 16S rRNA gene sequences (39, 40). The ASVs of chloroplast and mitochondria were removed, and then the data set was rarefied at the minimal read counts among samples (3,250 reads).

The soil bacterial community analyses were conducted and visualized using the MARco (41), vegan (42), and pheatmap (43) packages in R software (44). A Kruskal-Wallis test and Dunn’s *post hoc* test were used for all statistical analyses of group comparisons with a significance level of α = 0.05, and the *P* values were adjusted with a false discovery rate (FDR). Alpha diversity indices were estimated by richness, Shannon’s index, Simpson’s index, and Chao1 index. Beta diversity of microbial communities was measured by Bray-Curtis dissimilarity using a principal coordinates analysis (PCoA), and heterogeneity was tested using ADONIS and ANOSIM tests.

### Data availability.

The original data set presented in the study is publicly available. These data can be found at NCBI under BioProject accession number: PRJNA826673.
