# Supplementary material for: Soil Bacterial Community May Offer Solutions for Ginger Cultivation
Source: Microbiol Spectr. 2022 Sep 13;10(5):e01803-22. doi: 10.1128/spectrum.01803-22 (PMC9603371; doi:10.1128/spectrum.01803-22)
Supplement: Supplemental file 1 — Fig. S1 to S4; Table S1. Download spectrum.01803-22-s0001.pdf, PDF file, 1.4 MB [file spectrum.01803-22-s0001.pdf]

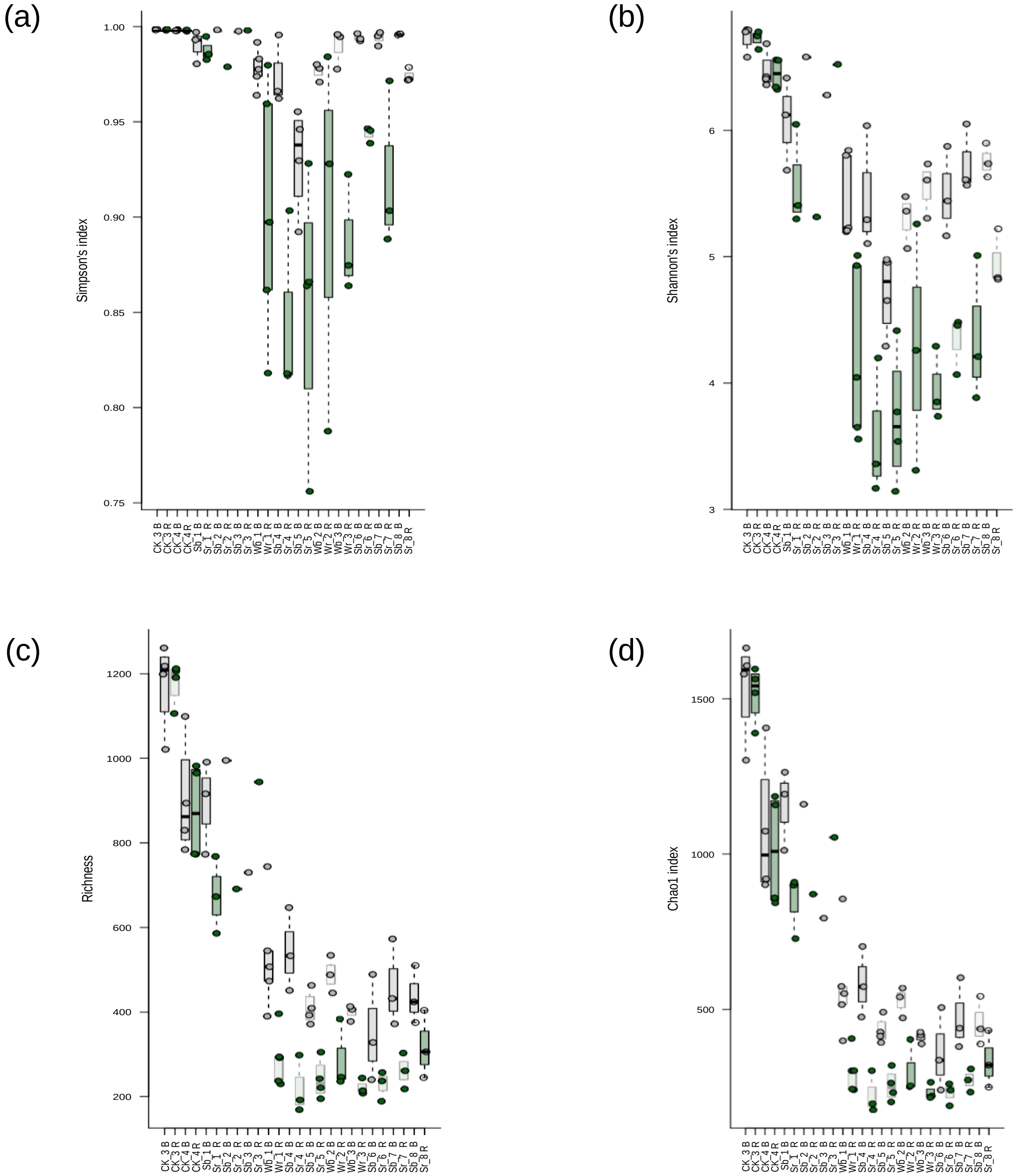

Figure S1. Alpha diversity indices of bacterial communities in each sample from bulk and rhizome parts of healthy and diseased soils. The grey color indicates the samples of bulk soil, and the green color indicates the samples of rhizome soil. CK indicates control soil samples, Sr is soil with soft rot disease, and Wr is soil with wilt disease. Y axis in (a) is Simpson index; in (b) is Shannon's index; in (c) is Richness; in (d) is Chao1 index.

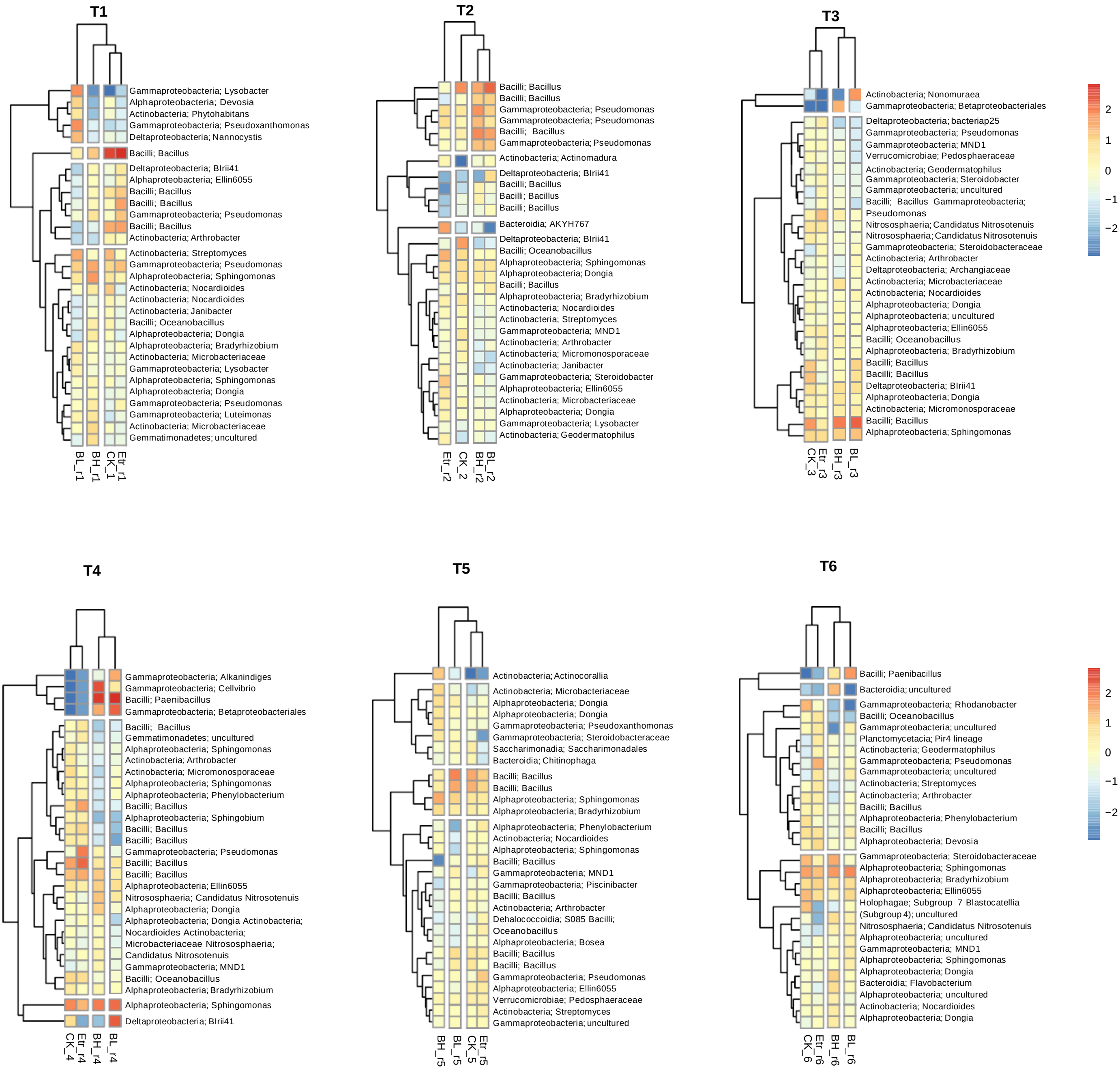

Figure S2. The relative abundance of the top 30 ASVs in rhizome parts for four treatments at each sampling time. CK indicates ginger soil without any treatment, BL is low amounts of *B. velezensis*, BH is high amounts of *B. velezensis*, and Etr is the Etridiazole treatment. At different time points (T1-T6), there is a remarkable similarity between the BH and BL bacterial compositions, and a similarity between the Etr group and CK group bacterial compositions.

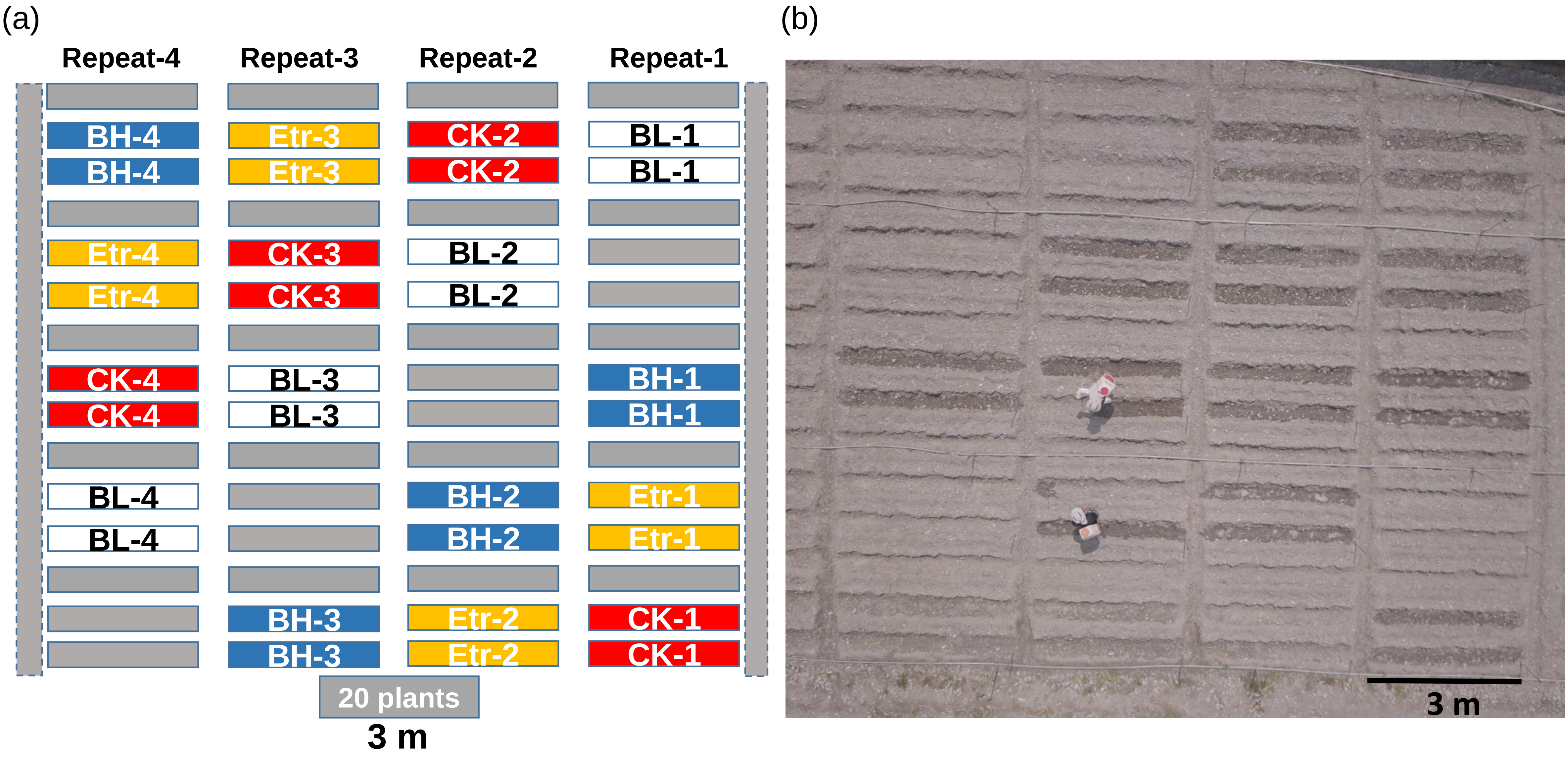

Figure S3. The experimental design for the fieldwork. Ginger plants were planted on March 29, 2019, using four treatments: BV138 200x dilution (BL group), BV138 25x dilution (BH group), Terrazole® (containing 25% etridiazole) 1500x dilution (Etr group), and a control (CK) irrigated with water. Each treatment plot was 3 meters long and 0.3 meters wide with two rows planted with 40 ginger seed rhizomes. Each treatment was conducted with four replicates and arranged by Randomized Complete Block Design (RCBD).

Date	Apr			May		Jun					Jul						Aug				Sept			Oct	Nov	Jan
	1	3	26	2	14	3	5	16	18	30	1	3	18	20	26	31	16	21	23	26	11	15	28	18	13	6
Microbial reagent																										
Fungicide & pesticide																										
Fertilizer																										
Herbicide																										
Sampling Time		T0	T1			T2					T3									T4					T5	T6

Figure S4. Timetable of microbial reagent and chemicals applied during the treatment, as well as the sampling time. The colors indicate different applications. Purple indicates the sampling time with respective timepoint.

Supplementary table 1. Production information on the gingers in the study.

Treatment	Duplicate1	Duplicate2	Duplicate3	Duplicate4	Average	SD	SE	Production (kg)	Ratio to Control
BL	27.68	25.15	27.18	39.59	29.90	6.55	3.28	29.90 ± 3.28 ab	1.07
BH	35.28	39.36	42.88	29.04	36.64	5.94	2.97	36.64 ± 5.94 a	1.31
Etr	25.28	30.49	10.88	14.73	20.34	9.10	4.55	20.34 ± 4.55 b	0.73
Control	29.30	25.23	23.87	33.48	27.97	4.34	2.17	27.97 ± 2.17 ab	1.00
